# Isotretinoin‐Associated Myopathy With Normal Creatine Kinase (CK) Levels: EMG‐Based Diagnosis at Low Dose—A Case Report

**DOI:** 10.1002/ccr3.71210

**Published:** 2025-10-07

**Authors:** Mehran Mahyar, Roya Jahanbazi, Ali Khorram, Soheil Shahramirad, Mohammadreza Vataniman

**Affiliations:** ^1^ Department of Neurology, Faculty of Medicine Hormozgan University of Medical Sciences Bandar Abbas Iran; ^2^ Department of Medical Sciences Yazd.C., Islamic Azad University Yazd Iran; ^3^ Student Research Committee, Faculty of Medicine Hormozgan University of Medical Sciences Bandar Abbas Iran; ^4^ Department of Medical Sciences Sha.C., Islamic Azad University Shahrood Iran

**Keywords:** adverse effects, chemically induced, creatine kinase (CK), electromyography (EMG), isotretinoin, myopathy

## Abstract

Isotretinoin can cause toxic myopathy even at low doses with normal CK levels; proximal muscle weakness may be the only sign. EMG is essential for diagnosis, emphasizing the need for clinical vigilance and personalized assessment in patients with unexplained muscle symptoms on isotretinoin.

## 1 Introduction

Isotretinoin, a synthetic derivative of vitamin A, is one of the most effective systemic treatments for severe and refractory acne [1]. It modulates key cellular pathways, particularly apoptosis and immune regulation, exerting broad effects on various tissues including skin, liver, and muscle [2]. While its dermatologic and psychiatric adverse effects are well established, musculoskeletal complications are increasingly recognized [3]. Myalgia and elevated creatine kinase (CK) levels are reported in a substantial proportion of patients; however, rare cases may develop myopathy without significant CK elevation, underscoring the diagnostic value of complementary tools like electromyography (EMG) [4].

Isotretinoin‐induced toxic myopathy is uncommon but clinically relevant, with presentations ranging from mild proximal weakness to severe necrotizing myositis [5]. Although most cases occur early in therapy, delayed‐onset presentations may manifest with nonspecific symptoms that complicate timely diagnosis [6]. In this report, we present a rare case of delayed‐onset, low‐dose isotretinoin‐induced myopathy with normal CK levels and confirmatory EMG findings. The case highlights the importance of considering this adverse effect in the differential diagnosis of unexplained proximal muscle weakness and emphasizes the critical role of EMG in diagnosing drug‐induced myopathies, particularly when laboratory markers are inconclusive [7].

## 2 Case History/Examination

A 26‐year‐old woman with no prior medical history presented with a five‐week history of progressively worsening weakness in all four limbs. She reported increasing difficulty climbing stairs and raising her arms to brush her hair. In addition, she described fatigue and pain in the jaw muscles during prolonged speaking or eating, with a clear pattern of diurnal fluctuation. She denied dysphagia, sensory disturbances, or systemic complaints.

Because of the jaw involvement and fluctuation of symptoms, the differential diagnosis included neuromuscular junction disorders such as myasthenia gravis (MG). Therefore, a myasthenic panel—including anti‐AChR and anti‐MuSK antibodies—was requested, both of which returned negative. Furthermore, due to her young age and presentation with proximal myopathy, rheumatologic conditions were also considered. A comprehensive autoimmune panel including ANA, anti‐dsDNA, and 25(OH) vitamin D was performed and yielded normal results, effectively ruling out rheumatologic or autoimmune etiologies.

The patient had been taking oral isotretinoin (10 mg, twice weekly) for moderate acne over the past five months. Other medications included sertraline 50 mg/day and alprazolam 0.5 mg/day. She denied any substance use or family history of neuromuscular disease.

## 3 Differential Diagnosis, Investigations and Treatment

Neurological examination revealed symmetrical proximal muscle weakness (deltoid and iliopsoas 3/5, biceps and quadriceps 4/5), with preserved distal strength and normal tone. Gower's sign was positive. No muscle atrophy, fasciculations, or tremors were noted. Cranial nerves were grossly intact, except for mild weakness in the trapezius and sternocleidomastoid muscles. Sensory and cerebellar exams were unremarkable. Deep tendon reflexes were symmetrical at 2+, and plantar responses were flexor bilaterally.

Routine laboratory investigations—including CBC, electrolytes, renal and hepatic panels, thyroid function, ferritin, calcium, phosphorus, magnesium, and vitamin D (24.4 ng/mL)—were within normal ranges. Serum creatine kinase (CK) was 109 IU/L (normal), while LDH was mildly elevated at 256 IU/L. In drug‐induced myopathy, CK levels can often remain within normal limits; hence, normal CK does not exclude myopathy.

Electromyography and nerve conduction studies showed a classic myopathic pattern involving proximal muscles including the deltoid, biceps, iliopsoas, and rectus femoris, with short‐duration, low‐amplitude, polyphasic motor unit potentials and early recruitment. No spontaneous activity or signs of denervation were observed. Distal muscle groups appeared unaffected. These findings confirmed a symmetric, proximal, subacute myopathy (see Table 1 for detailed EMG findings). An MRI of the brain and cervical spine, ordered by the neurology team, was normal.

**TABLE 1:** Electromyography (EMG) findings by muscle.

Muscle	Pattern	Spontaneous activity	MUAP^a^	Recruitment
Deltoid L^b^	Myogenic	None	Polyphasic	Easy
Deltoid R^c^	Myogenic	None	Polyphasic	Easy
Biceps L^d^	Myogenic	None	Polyphasic	Easy
Biceps R^e^	Myogenic	None	Polyphasic	Easy
Extensor indicis L^f^	Normal	None	Polyphasic	Easy
Extensor indicis R^g^	Normal	None	Polyphasic	Easy
Iliopsoas L^h^	Myogenic	None	Polyphasic	Easy
Rectus femoris L^i^	Myogenic	None	Polyphasic	Easy
Rectus femoris R^j^	Myogenic	None	Polyphasic	Easy
Gastrocnemius (med) L^k^	Normal	None	Full MUP	Full
Gastrocnemius (med) R^l^	Normal	None	Full MUP	Full
Tibialis posterior L^m^	Normal	None	Full MUP	Full

^a^ Motor unit action potential.

^b^ Left deltoid muscle.

^c^ Right deltoid muscle.

^d^ Left biceps muscle.

^e^ Right biceps muscle.

^f^ Left extensor indicis muscle.

^g^ Right extensor indicis muscle.

^h^ Left iliopsoas muscle.

^i^ Left rectus femoris muscle.

^j^ Right rectus femoris muscle.

^k^ Left medial gastrocnemius muscle.

^l^ Right medial gastrocnemius muscle.

^m^ Left tibialis posterior muscle.

## 4 Conclusion and Results (Outcome and Follow‐Up)

The temporal correlation with isotretinoin use, absence of alternative etiologies, and confirmatory EMG findings supported the diagnosis of isotretinoin‐induced toxic myopathy. Following discontinuation of isotretinoin, the patient experienced rapid clinical improvement within three weeks. Gower's sign resolved, and full muscle strength and function were restored. No corticosteroids or other treatments were required.

This case illustrates a subacute, symmetric proximal quadriparesis caused by isotretinoin‐induced toxic myopathy. It underscores the importance of considering retinoid‐associated muscle toxicity even at low doses and highlights the diagnostic utility of EMG in confirming myopathy, particularly when CK levels are normal.

## 5 Discussion

Isotretinoin, a 13‐cis‐retinoic acid derivative, is a widely prescribed systemic retinoid for the treatment of moderate to severe acne, with mechanisms of action believed to involve sebocyte apoptosis, immunomodulation, and alterations in lipid metabolism, largely through activation of Forkhead box O (FoxO) transcription factors [8]. While its dermatologic and psychiatric side effects are well‐documented, musculoskeletal toxicities—including myalgia, arthralgia, sacroiliitis, enthesitis, neuropathies, and toxic myopathies—have been increasingly recognized [3]. Although isotretinoin‐induced myopathy remains rare, it should not be underestimated, as prior studies have described cases of rhabdomyolysis, multifocal myositis, and proximal weakness, often accompanied by elevated creatine phosphokinase (CK) levels and occurring early in the treatment course [6]. In contrast, our patient exhibited a distinct clinical trajectory; despite five months of low‐dose therapy, she developed progressive proximal quadriparesis—beginning five weeks prior to presentation—with completely normal CK (109 IU/L), and while most literature associates symptomatic muscle injury with CK elevations. Isotretinoin toxicity creates a critical need for clinicians to vigilantly monitor muscle symptoms during therapy [9]. Recent reports—including our own—highlight that CPK can remain within normal limits in true cases of drug‐induced myopathy, aligning with newer observations suggesting that CPK alone lacks sufficient sensitivity for early or low‐grade muscular toxicity [10].

Further divergence from typical cases lies in the timing and severity, as most published reports describe symptom onset within 30 to 60 days of therapy initiation, typically in the setting of physical exertion or high‐dose regimens, whereas our patient had a delayed presentation and lacked any precipitating physical stressor. Her rapid and complete recovery after isotretinoin withdrawal without corticosteroids or immunosuppressants contrasts with other reports requiring prolonged immunotherapy, indicating a more benign and reversible form of toxicity in our case [5]. Moreover, the exclusive involvement of proximal muscles with preserved distal strength and reflexes diverges from more diffuse or asymmetric patterns described elsewhere. Electromyography (EMG) findings were critical in our case; despite unremarkable laboratory parameters, EMG revealed a clear myopathic pattern in the deltoid, biceps, iliopsoas, and rectus femoris muscles, characterized by short‐duration, low‐amplitude, polyphasic motor unit potentials with early recruitment and no spontaneous activity. These findings, restricted to proximal muscle groups, stand in contrast to reports of diffuse or multifocal involvement, particularly those associated with systemic symptoms or concurrent autoimmune features [6, 11]. In our case, evaluation for myasthenia gravis was prompted by the presence of jaw fatigue and diurnal fluctuation, and rheumatologic tests were pursued due to the patient's young age and proximal weakness pattern; both workups returned negative results. Autoimmune and myasthenic panels were negative, the brain and cervical MRI were normal, and no sensory involvement or systemic inflammation was observed, helping rule out inflammatory, neuromuscular junction, and neurogenic causes.

In addition to clinical and electrodiagnostic contrast, mechanistic differences are also worth noting, as prior studies propose FoxO hyperactivation, mitochondrial dysfunction, and sarcolemmal membrane instability via MMP‐2 as plausible pathways for retinoid‐induced muscle injury [12]. These mechanisms may underlie both inflammatory and degenerative changes reported in other cases, while the localized, rapidly reversible presentation in our patient supports a predominantly non‐inflammatory, dose‐independent myotoxic effect—possibly reflecting a threshold‐based mitochondrial or metabolic insult rather than immune‐mediated damage. Furthermore, many published cases of isotretinoin‐induced muscle toxicity have been associated with significantly elevated CK levels—often exceeding 1000 IU/L—and accompanied by myoglobinuria or systemic malaise, whereas the absence of such laboratory derangements in our patient highlights the importance of maintaining high clinical suspicion, particularly when proximal weakness arises in the absence of biochemical evidence [13, 14]. Our case supports recommendations that serum enzymes should not be relied upon solely and that EMG serves as an essential diagnostic adjunct, especially when symptoms are subtle and other causes have been excluded.

Lastly, our case emphasizes the necessity of personalized drug monitoring. Although musculoskeletal toxicity is considered dose‐dependent, our patient experienced clinically significant myopathy despite a low cumulative dose, suggesting that individual susceptibility—perhaps modulated by genetic or metabolic factors—may play a larger role than previously appreciated. Clinical vigilance should therefore extend beyond standard risk profiles, particularly in younger patients presenting with unexplained proximal weakness. In conclusion, this case contributes a unique variant of isotretinoin‐induced toxic myopathy to the literature: delayed‐onset, CPK‐normal, proximally localized, and fully reversible upon drug cessation. It underscores the need for heightened clinical awareness, the diagnostic value of EMG when laboratory findings are inconclusive, and the importance of tailored patient evaluation even in low‐risk dosing scenarios, with broader documentation of such atypical presentations aiding in refining diagnostic criteria and guiding future pharmacovigilance.

## Author Contributions

**Mehran Mahyar:** methodology, project administration, supervision. **Roya Jahanbazi:** formal analysis, writing – review and editing. **Ali Khorram:** investigation, methodology, writing – original draft. **Soheil Shahramirad:** data curation, writing – original draft. **Mohammadreza Vataniman:** writing – original draft, writing – review and editing.

## Consent

Written informed consent was obtained from the patient for publication of this case report and any accompanying images.

## Conflicts of Interest

The authors declare no conflicts of interest.

## Data Availability

Data sharing is not applicable to this article as no datasets were generated or analyzed during the current study.
